# Music Therapy in the Treatment of Dementia: A Systematic Review and Meta-Analysis

**DOI:** 10.3389/fmed.2020.00160

**Published:** 2020-05-19

**Authors:** Celia Moreno-Morales, Raul Calero, Pedro Moreno-Morales, Cristina Pintado

**Affiliations:** ^1^Department of Inorganic Chemistry, Organic Chemistry and Biochemistry, Faculty of Environmental Sciences and Biochemistry, University of Castilla-La Mancha, Toledo, Spain; ^2^School of Nursing and Physiotherapy, University of Castilla-La Mancha, Toledo, Spain; ^3^Regional Centre for Biomedical Research, University of Castilla-La Mancha, Albacete, Spain

**Keywords:** systematic review, meta-analysis, dementia, music therapy, cognitive function, quality of life, depressive state

## Abstract

**Background:** Dementia is a neurological condition characterized by deterioration in cognitive, behavioral, social, and emotional functions. Pharmacological interventions are available but have limited effect in treating many of the disease's features. Several studies have proposed therapy with music as a possible strategy to slow down cognitive decline and behavioral changes associated with aging in combination with the pharmacological therapy.

**Objective:** We performed a systematic review and subsequent meta-analysis to check whether the application of music therapy in people living with dementia has an effect on cognitive function, quality of life, and/or depressive state.

**Methods:** The databases used were Medline, PubMed Central, Embase, PsycINFO, and the Cochrane Library. The search was made up of all the literature until present. For the search, key terms, such as “music,” “brain,” “dementia,” or “clinical trial,” were used.

**Results:** Finally, a total of eight studies were included. All the studies have an acceptable quality based on the score on the Physiotherapy Evidence Database (PEDro) and Critical Appraisal Skills Program (CASP) scales. After meta-analysis, it was shown that the intervention with music improves cognitive function in people living with dementia, as well as quality of life after the intervention and long-term depression. Nevertheless, no evidence was shown of improvement of quality of life in long-term and short-term depression.

**Conclusion:** Based on our results, music could be a powerful treatment strategy. However, it is necessary to develop clinical trials aimed to design standardized protocols depending on the nature or stage of dementia so that they can be applied together with current cognitive-behavioral and pharmacological therapies.

## Key Points

- Music therapy is used as a treatment for the improvement of cognitive function in people with dementia.
- The intervention based on listening to music presents the greatest effect on patients with dementia followed by singing.
- Music therapy improved the quality of life of people with dementia.
- Music has a long-term effect on depression symptoms associated with dementia.

## Introduction

Approximately 50 million people worldwide have dementia, and it is projected to almost triple by 2050 (1). Dementia is an overall term for diseases and conditions characterized by progressive affectation of cognitive alterations, such as memory and language, as well as behavioral alterations including depression and anxiety (2, 3). In order to ameliorate the symptoms of dementia, different intervention approaches, both pharmacological and non-pharmacological, have been trialed. Pharmacological interventions, such as acetylcholinesterase inhibitors, are mainly aimed to treat cognitive symptoms but without avoiding the course of the disease. Unfortunately, these therapies have limited effect on alleviating behavioral and psychological symptoms of dementia (2, 4). On the other hand, non-pharmacological interventions can provide complementary therapy, offering versatile approaches to improve outcomes for people living with dementia and minimize behavioral occurrences as well as to improve or sustain quality of life (2, 5–9). There are many types of non-pharmacological approaches, such as psychosocial and educational therapies (either with individuals or in groups) and physical or sensorial activities (music, therapeutic touch, and multisensory stimuli) (7, 10–12). In particular, music therapy is thoroughly used in daily clinical practice in case of dementia (13, 14). Many authors emphasize the positive effects of music on the brain. In this sense, several studies showed that people with dementia enjoy music, and their ability to respond to it is preserved even when verbal communication is no longer possible. These studies claimed that interventions based on musical activities have positive effects on behavior, emotion and cognition (2, 15, 16). Therefore, studying and playing music alter brain function and can improve cognitive areas, such as the neural mechanisms for speech (17), learning, attention (18), and memory (19). Music can also activate subcortical circuits, the limbic system, and the emotional reward system, provoking sensations of welfare and pleasure (14). In this regard, long-term musical training and learning of associated skills can be a strong stimulus for neuroplastic changes, in both the developing brain and the adult brain. These findings suggest the great capacity of music to enhance cerebral plasticity (13, 16, 20). Contrariwise, there are studies that question the specific effect of music therapy on people with dementia (21). With this background, the aim of this study is to analyze from an unbiased approach the effect of music therapy on the cognitive function, quality of life, and/or depressive state in people living with dementia.

## Methods

### Search Strategy and Selection Criteria

**Figure 1:**
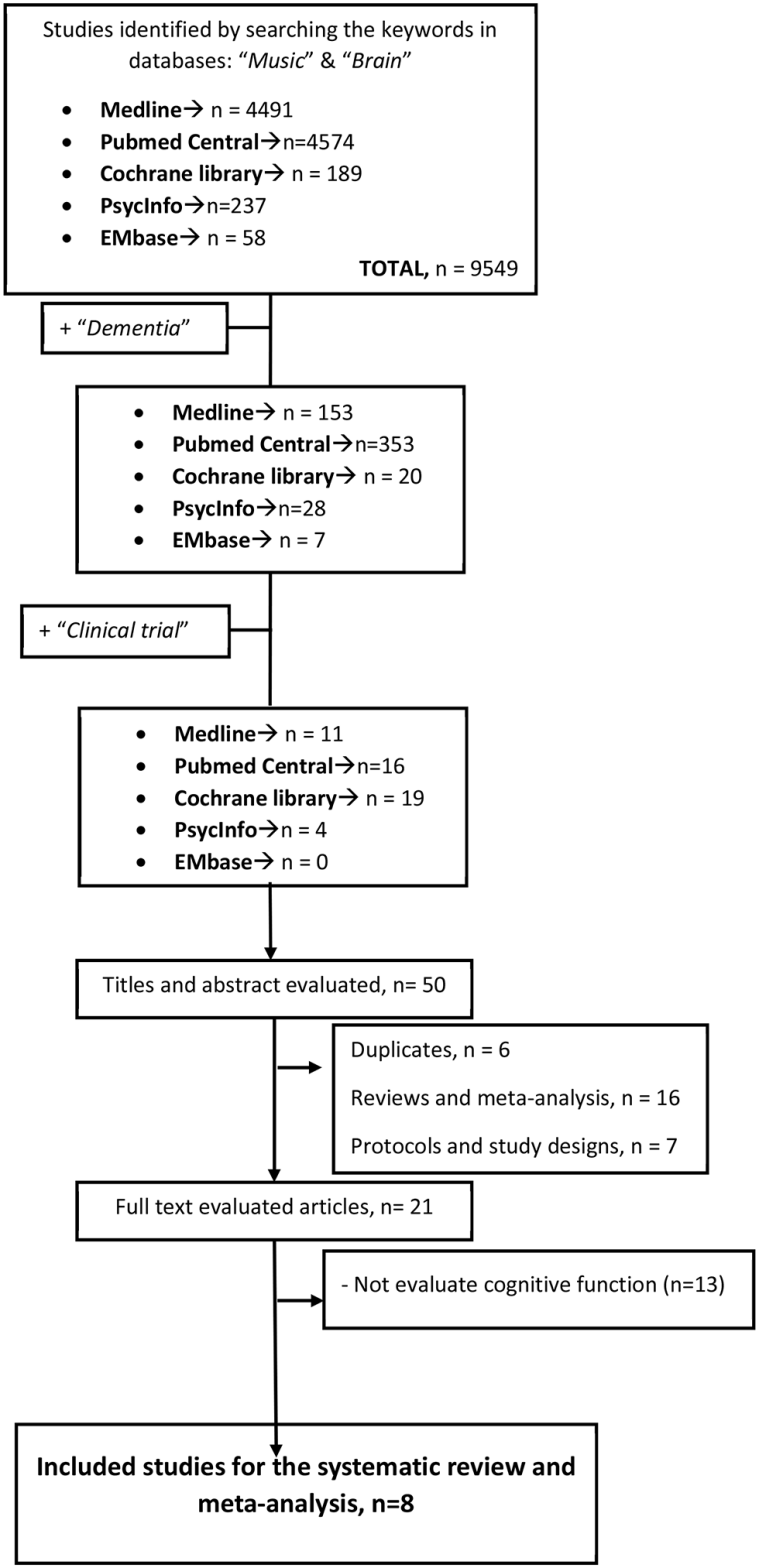
Flow of studies through the review process for systematic review and meta-analysis.

A systematic review was conducted following the recommendations of the Preferred Reporting Items for Systematic Reviews and Meta-analyses (PRISMA) (Figure 1 and Searching procedure of Supplementary Data) (22). An independent literature search was conducted across Medline, PubMed Central, Embase, PsycINFO, and Cochrane library databases. We carried out the systematic review of the literature following a series of criteria as detailed below.

Initially, the search began with the terms “brain” and “music.” Later, “dementia” was added, and finally, “clinical trial” was included. The search period used was from 1990 to present. Next, a more in-depth study of selected trials was carried out. Duplicate studies were removed. All studies that compared any form and method of musical intervention with an intervention without music were evaluated. Lastly, those studies that were systematic analysis, reviews, and study protocols and those which do not evaluate cognitive function were excluded. All the trials chosen were designed as randomized controlled trials (RCTs).

### Data Collection, Extraction, and Quality Assessment

Two authors (CMM and PMM) independently assessed publications for eligibility. Discrepancies or difficulties were discussed with a third review author (CP). Data were collected independently using a standardized data extraction form in order to summarize the characteristics of the studies and outcome data (23).

From each individual study, we extracted baseline information: publication and year, study design, participants (number, age, and sex ratio), Mini-Mental Status Examination (MMSE) score, and Clinical Dementia Rating (CDR) (clinical evaluation of dementia) when possible, as well as the design of each individual study (intervention method, frequency, duration, and time of evaluation of the results) (Table 1).

**Table 1:** Characteristics of the studies.

	**SUBJECTS**					**DESIGN**		
	**Study** **design**	**Participants** **(age, sex & percentage of women)**	**Type of participants** **(population)**	**Baseline** **MMSE score**	**CDR**	**Intervention;** **kind of music**	**Intervention control** **(minutes/per week/ weeks)**	**Main** **Findings**
Särkämö et al. (24)	RCT	*N* = 89 SG: 27 → 59.3% 78.5 ± 10.4 MLG: 29 → 89.6% 79.4 ± 10.1 CG:28 → 64.3% 78.4 ± 11.6	People living with mild to moderate dementia, without serious psychiatric illness or substance abuse	SG: 19.08 ± 2.5 MLG: 15.7 ± 2.26 CG: 18.57± 2.64	SG: 1.0 ± 0.6 MLG: 1.6 ± 0.5 CG: 1.1 ± 0.5	Active: sing Passive: listen to music Control group No significant differences in clinical medication between groups	90 min once a week for 10 weeks	Improvement of mood, orientation and memory Soft effect on attention, executive function, and general cognition.
Särkämö et al. (25)	RCT	*N* = 89	People living with mild to moderate dementia	SG F1: 22.9 ± 3.4 F2: 21.0 ± 4.8 MLG F1: 21.0 ± 4.6 F2: 18.8 ± 4.5 CG F1: 21.5 ± 4.8 F2: 19.6 ± 6.3	-	Active: sing Passive: listen to music Control group No significant differences in clinical medication between groups	90 min once a week for 10 weeks	MLG had benefits in the behavioral alterations of depression, while the SG showed specific benefits in physical signs of depression.
Doi et al. (26)	RCT	*N* = 201 Average age 76.0, 52% women DG: 67 → 50.7% 75.7 ± 4.1 IMG: 67 → 58.2% 76.2 ± 4.6 CG: 67 46.3% 76.0 ± 4.9	Older adults living with mild cognitive impairment	DG: 26.29 ± 2.6 IMG: 26.36 ± 2.1 CG: 25.4 ± 2.3	-	Interactive, two cognitive programs of leisure activities: (1) Dance (2) Musical instruments	60 min per week for 40 weeks	Long-term cognitive leisure activities involving dance or playing musical instruments improved memory and general cognitive function
Han et al. (27)	RCT	*N* = 64 Age = 76.22 ± 5.84 62.5% women MG: 32 75.63 ± 6.3 68.8% CG: 32 76.81 ± 5.36 56.3%	Older people living with mild cognitive impairment or mild dementia	MG: 23.45 ± 4.76 CG: 22.87 ± 4.68	Patients presenting 0.5 of CDR: 47 (73.4%)	MG: Multimodal cognitive improvement therapy (MCET) CG: Mock Therapy No significant differences in clinical medication between groups	2 treatment phases of 8 weeks separated by a period of 4 weeks. Total: 20 weeks	MCET improves cognition, behavior and quality of life
Ceccato et al. (28)	RCT	*N* = 51 MLG: 27 → 77.8% 85.5 ± 5.9 CG: 23 → 82.6% 87.2 ± 7.1	Elderly people living with mild and moderate dementia	MLG:16.93 ± 3.66 CG:16.39 ± 3.90	-	Sound Training for Attention and Memory STAM-Dem (listening)	Two weekly session of 45 min for 12 weeks	General improvement in the results of the experimental group
Lyu et al. (29)	RCT	*N* = 192 SG: 97 → 58.7% 68.9 ± 7.1 CG:95 → 58.9% 69.9 ± 7.9	People living with mild, moderate or severe Alzheimer- type dementia	SG:13.45 ± 3.66 CG: 13.22 ± 4.01	-	Sing vs usual care	Music therapy practiced in groups. twice daily. 30–40 min per session for 3 months (12 weeks)	Effectiveness in enhancing cognitive function and mental well being
Chu et al. (30)	RCT	*N* = 100 MG:49 CG:51 53% 82 ± 6.80	People living with Moderate (62%), mild (17%) and severe (21%) dementia	MG12.80 ± 6.15 CG: 13.76 ± 5.36	-	Playing an instrument, Sing and listening to music	12 sessions of group music therapy (two 30-min sessions per week for 6 weeks)	Music intervention appeared to reduce depression and delayed the deterioration of cognitive functions, particularly short-term recall function.
Guétin et al. (31)	RCT	*N* = 30 MLG:15 → 86.7% CG:15 → 60% 75–90	People living with mild to moderate Alzheimer- type dementia	MLG:19.8 ± 4.4 CG: 20.7 ± 3.4	-	Listening to music	Session once a week for 16 weeks	No significant differences between MLG and CG

*F1, follow-up 1 (short term); F2, follow-up 2 (long term); RCT, randomized clinical trial; CG, control group; AD, Alzheimer's disease; SG, singing group; MLG, music listening group; DG, dancing group; IMG, instrumental music group; MG, Music group*.

In addition, at the beginning of the study, we assessed the quality of meta-analysis-included studies using the Physiotherapy Evidence Database (PEDro) scale and the Critical Appraisal Skills Program (CASP) scale (Supplementary Tables 1, 2 of the Supplementary Data) (23, 32, 33).

### Outcome Measures

The primary outcome defined to be compared was cognitive function evaluated through MMSE (34), Alzheimer's Disease Assessment Scale-cognitive subscale (ADAS-Cog) (35), Revised Memory and Behavior Problems Checklist (RMBPC) (36), or Immediate and Deferred Prose Memory test (MPI and MPD, respectively) (37). Other comparative results, named as secondary outcomes, were quality of life, assessed through Quality of Life in Alzheimer's Disease (QOL-AD) (38), and depression, evaluated through Cornell–Brown Scale for Quality of Life in Dementia (CBS) and Geriatric Depression Scale (GDS) (39, 40).

### Statistical Analysis: Meta-Analysis

First, a comparison was made using the random-effects model. All outcomes were continuous variables [mean ± standard deviation (SD) of the change in the score before and after the therapy in the different diagnostic tests], and the standardized mean difference (SMD) was analyzed. All the analyses were carried out considering a confidence interval (CI) of 95%. Statistical heterogeneity was also tested by *I*^2^. *I*^2^ <25% was identified as low heterogeneity (41, 42). Finally, the publication bias was evaluated using funnel plot graphs (43, 44). To further investigate the heterogeneity, meta-regression and subgroup analyses were performed to assess the primary outcome data and associations according to the method of intervention (interactive and passive), trial period, number of sessions per week, and effect of evaluation method used. The *P* values in the meta-regression revealed the overall significance of the influence factors.

Meta-analysis, heterogeneity study, and graphical representations were performed using R with the Metafor package (44). To digitize graphics and obtain numerical data from those trials that did not provide them, the GetData Graph Digitizer program (Getdata-graph-digitizer.com) was used.

## Results

### Baseline Characteristics

Results of initial search and exclusions are shown in Figure 1. A thorough reading of each article was carried out, and a summary of each of them is shown in Table 1. Therefore, we finally stayed for the systematic review and meta-analysis with eight articles. The size of the studies was between 30 and 201 subjects, with a total of 816 subjects with mild to severe dementia, assigned randomly to both the intervention and control groups. All the people in the trials stayed in nursing homes or hospitals. Särkämö et al. divided the participants into three groups, an active group that sang, a passive group that listened to music, and a control group (24, 25). On the other hand, Doi et al. evaluated two cognitive programs of leisure activities: dancing and playing musical instruments (26). Furthermore, Han et al. tested a multimodal cognitive improvement therapy (MCET) consisting of cognitive training, cognitive stimulation, reality orientation, physical, reminiscence, and music therapy against a sham therapy without music (27). In this line, Ceccato et al. tried the program Sound Training for Attention and Memory in Dementia (STAM-Dem), a manualized music-based protocol designed to be used in the rehabilitation of cognitive functions in people with dementia. Those in the control group continued with the normal “standard care” provided (28). While Lyu et al. compared the effect of singing on cognitive function and mood, Chu et al. assessed a protocol that includes playing an instrument, dancing, and listening to music. The effect size of all those studies reveals a general improvement in the results of the experimental group (29, 30). Finally, Guétin et al. did not find a significant difference between the experimental and control groups when evaluating the cognitive function after an 18-month therapy based on listening to music (31).

All the studies had an acceptable quality as confirmed after applying the PEDro and CASP scales (Supplementary Tables 1, 2, respectively, of the Supplementary Data).

In case of medication (dementia, antipsychotic, and antidepressant medication and sedative or sleeping medication), it must have been stable prior to the trial. Since participants were randomized, there were no significant differences between the control and music-treated groups with regard to medication. Likewise, there were no significant differences between groups in the dementia severity and/or demographic variables.

### Efficacy of Musical Intervention in Cognitive Function

**Figure 2:**
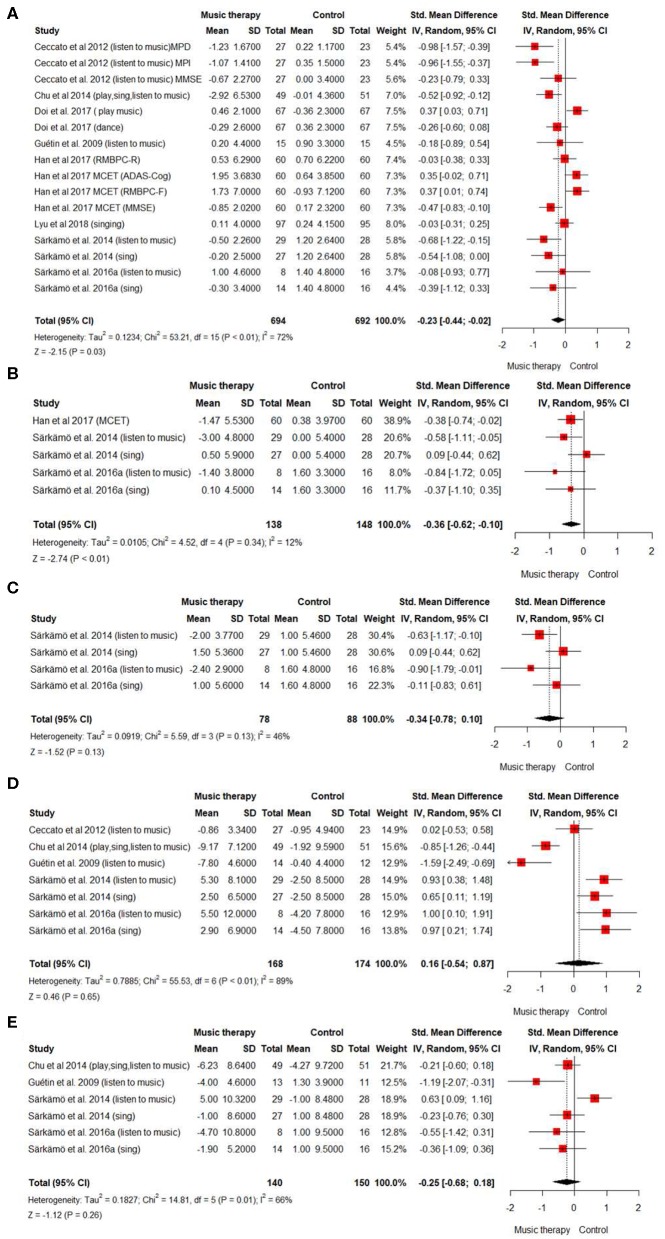
Summary of efficacy of music intervention on cognitive function and secondary outcomes. Forest plot. Overall efficacy of music intervention in people with dementia **(A)** on cognitive function. **(B)** on quality of life. **(C)** on quality of life of people after 6 months of treatment. **(D)** on depressive state **(E)** on depressive state after 6 months.

Figure 2 summarizes the relevant results of the quantitative synthesis of the effect of music therapy for people living with dementia. First, we evaluated the effect of music therapy on cognitive function by analyzing eight studies (816 cases) (Figure 2A). In the random-effects model, SMD was −0.23 (95% CI: −0.44, −0.02), which suggested that musical intervention could be beneficial to improve cognitive function in people living with dementia. However, the trials showed very high heterogeneity [*I*^2^ value = 72% (*P* < 0.0001)].

Subgroup analyses and meta-regression were used to further explore this source of heterogeneity (Table 2). Two significant sources of heterogeneity were detected: the trial period and the intervention method (coefficient = 7.43, *P* = 0.006 and coefficient = 3.981; *P* = 0.046, respectively). Interestingly, we observed that shorter intervention periods (<20 weeks) and passive interventions methods (listening to music) had greater effect on people living with dementia than longer intervention periods or interactive interventions, such as singing and dancing (Figure 2A; Table 2). On the other hand, to play an instrument does not seem to have a positive effect on cognitive function. Nevertheless, it appears to be effective when it is combined with singing and listening to music, without improving the effect of just listening to music (Figure 2A). The funnel plot on the publication bias across cognitive studies appeared symmetrically low (Supplementary Figure 1 of the Supplementary Data).

**Table 2:** Meta-regression for the effect of music intervention vs. control on cognitive function.

			**Subgroup analysis**			**Meta-regression**		
**Outcome or measure**		**k**	**SMD (95% CI)**	***P* value**	**I^**2**^ (%)**	**Coefficient (95% CI)**	***P* value**	**R^**2**^ (% of heterogeneity accounted for)**
	Overall	16	-0.2311 [-0.4416;−0.0206]^*^	0.0314	71.8	-	-	-
Intervention length	<20 weeks	9	-0.4751 [-0.7235;−0.2267]^*^	0.0002	50.7	7.429	**0.0064**^**a**^	34.4
	>20 weeks	7	0.0364 [-0.2323; 0.3050]	0.7908	70.9			
Intervention method	Interactive	6	−0.1899 [-0.4811; 0.1013]	0.2011	68.2	3.981	**0.0460**^**a**^	45.1
	Passive	6	-0.5648 [-0.8776;−0.2521]^*^	0.0004	33.6			
Intervention method	Dancing	1	-	-	-	0.182	0.6699	0.0
	Singing	3	−0.2335 [-0.5724; 0.1053]	0.1768	34.6			
	Listen to music	6	-0.5648 [-0.8776;−0.2521]^*^	0.0004	33.6			
	Playing music	1	-	-	-			
Sessions/week	1	9	−0.0889 [-0.3569; 0.1791]	0.5157	74.1	2.847	0.0915	9.4
	>1	7	-0.4432 [-0.7518;−0.1346]^*^	0.0049	58.9			
Evaluation method	MMSE	11	-0.2546 [-0.4593;−0.0498]^*^	0.0148	54.0	0.183	0.6691	0.0
	Others	5	−0.2029 [-0.7147; 0.3088]	0.4370	85.8			

* Results with significant differences.

a * Important source of heterogeneity*.

### Efficacy of Musical Intervention in Quality of Life

A meta-analysis about the quality of life of people living with dementia after the intervention with music therapy was designed. The analysis included three studies (286 cases). The results suggested that there was an effect on the quality of life of patients once the intervention is finished (SMD = −0.36, 95% CI: −0.62, 0.10) (Figure 2B). On the other hand, no significant effect of music therapy was observed when carrying out the analysis (two studies; 166 cases) of the quality of life of people living with dementia 6 months after the intervention (SMD = −0.34, 95% CI: −0.78, 0.10) (Figure 2C). The heterogeneity of the studies was small in the short-term analysis but >25 in the long term (*I*^2^ = 12 and *I*^2^ = 42, respectively).

Supplementary Figures 1B,C in the supplementary data represent the funnel plot about the quality of life measured after the intervention and 6 months later. Data indicate that there is no publication bias.

### Efficacy of Musical Intervention in the Depressive State

Finally, in order to evaluate the influence of music therapy on the depressive state associated with dementia, in both the short and long terms, we analyzed its effect when the intervention had just ended and 6 months after the treatment. The result of the meta-analysis (5 studies, 342 cases) suggested that there was no short-term effect on the depressive state of the patients (SMD = 0.16, 95% CI: −0.54, 0.87) (Figure 2D). However, when studying the depressive state of patients 6 months after the intervention to know if there is a long-term effect (4 studies, 290 cases), the result indicated that music therapy could have a positive effect on the depressive state of people living with dementia (SMD = −0.25, 95% CI: −0.68, 0.18) (Figure 2E). In both cases, the heterogeneity of the studies was high [*I*^2^ = 89% (*P* < 0.0001) in the short term; *I*^2^ = 66% (*P* < 0.01) in the long term]. The funnel plot of the depressive state after the intervention and about the depressive state at 6 months denotes that there is no publication bias (Supplementary Figures 1D,E in the Supplementary Data).

## Discussion

The main objective of this work was to study through systematic review and meta-analysis whether the application of music as a therapy has an effect on cognitive function, quality of life, and/or depressive state in a group of specific diseases such as dementia. Nowadays, there is a growing incidence of this pathology in the population (1), and therefore, it is necessary to develop treatments and activities to relieve its symptoms. In addition, there is not enough scientific evidence about the efficacy of music as a therapy on the cognitive and behavioral states of these patients.

Our results suggest that music therapy has a positive effect on cognitive function for people living with dementia. To reach that assumption, we performed a comprehensive systematic review that includes eight studies with 816 subjects. We observed that listening to music is the intervention type with the greatest positive effect on cognitive function. This could be explained because listening to music integrates perception of sounds, rhythms, and lyrics and the response to the sound and requires attention to an environment, which implies that our brain has many areas activated. Those events are linked to wide cortical activation (14, 15, 45). In addition, music training is a strong stimulus for neuroplastic changes. So music could decrease neuronal degeneration by enhancing cerebral plasticity and inducing the creation of new connections in the brain (46, 47). However, the heterogeneity presented by the different studies included in the meta-analysis does not allow us to reach reliable conclusions (*I*^2^^=^75%). This heterogeneity may be due to the design of each study, the difference in the type of intervention carried out, and the number of participants among other variables (41). Meta-regression showed that the intervention method, interactive or passive, is a significant source of heterogeneity accounting for 45.1% of the total heterogeneity detected (Table 2). We observed a significant effect on cognitive function in the passive intervention group (*P* = 0.0004). This result is in agreement with our previous analysis where listening to music has the greatest effect. Other sources of heterogeneity found when we analyzed the effect of music therapy on the cognitive function were the intervention length and the number of sessions per week (34.4 and 9.4%, respectively), the latter not being significant (Table 2). Based on the literature, there is a huge diversity in the scheduling of music treatment duration. In our case, sessions varied from 90 min once a week during 10 or 20 weeks to 60 min during 40 weeks. It seems that the length for the entire music intervention procedure might be a crucial element for successful results and seems to be associated with the intervention type (48–50). We observed that shorter intervention periods (<20 weeks) had a greater effect on people living with dementia than longer intervention ones. This finding is not enough to draw further conclusion due to the heterogeneity found. According to our results, although the number of sessions per week seems not to have an impact on music therapy effectiveness, a greater frequency of therapy seems to be of particular importance (48).

Xu et al. and Roman-Caballero et al. showed similar results in two meta-analysis studies conducted on musical intervention in cognitive dysfunction in healthy older adults (18, 23). In fact, as in our study, the level of heterogeneity found was also very wide. Van der Steen et al. also analyzed music-based therapeutic intervention on cognition in people with dementia (51). They found low-quality evidence that music-based therapeutic interventions may have little or no effect on cognition. Nevertheless, they did not analyze the effects in relation to the overall duration of the treatment, the number of sessions, and the type of music intervention.

After analysis of the secondary outcomes, music therapy surprisingly did not have a marked effect. Regarding quality of life, our data suggested a positive effect once the therapy is finished, but it was not durable after 6 months of music intervention. On the other hand, the study evaluating the effect of music therapy on the depressive state of people living with dementia showed no improvement in the state of these patients when they were evaluated after the intervention. However, if the depressive state was evaluated after 6 months from treatment, a shift in favor of music therapy was observed. This result suggests that the effects of music are not immediate and that the design of progressive and continuous interventions is necessary in order to obtain successful results as has also been discussed by Leubner and Hinterberger (49).

Xu et al. observed that, both in the analysis of the depressive state and in the quality of life, music therapy does not have a positive effect (23). These data corroborate the results obtained in the short term in our study. However, they did not measure the effects of long-term music therapy. Furthermore, Dyer et al. found that music as a non-pharmacological intervention improves behavioral and psychological symptoms of dementia but concluded that further research is required (2, 52). Van der Steen et al. also compared the effect music-based therapeutic intervention versus usual care or versus other activities on depression and emotional well-being (51). Likewise, at the end of treatment, they found low-quality evidence that the musical interventions may improve emotional well-being and quality of life.

Music is a pleasant stimulus, especially when it is adapted to one's personal preferences, and it can evoke positive emotions. Some studies have demonstrated that music therapy had an influence on levels of hormones such as cortisol. It also affects the autonomic nervous systems by decreasing stress-related activation (53, 54). At the same time, some studies suggest that music promotes several neurotransmitters, such as endorphins, endocannabinoids, dopamine, and nitric oxide. This implies that music takes part in reward, stress, and arousal processes (55). However, the lack of standardized methods for musical stimulus selection is a common feature in the studies we have reviewed. Additionally, the absence of a suitable control of the intervention to match levels of arousal, attentional engagement, mood state modification, or emotional qualities between participants may be a reason for the differences between studies (55). Furthermore, our results have likely been influenced by the type of test used to evaluate depression symptoms. Most studies used questionnaires that were based on self-assessment. However, it is unclear whether this approach is valid to detect changes regarding symptom improvement. Future approaches should add measurements of physiological body reactions, such as skin conductance and heart rate, for more objectivity (49).

## Conclusions

This study shows evidence with a positive trend supporting music therapy for the improvement of cognitive function in people living with dementia. Additionally, the study reveals a positive result for treatment of long-term depression, without showing an effect on short-term depression in these patients. Furthermore, music therapy seems to improve quality of life of people with dementia once the intervention is finished, but it does not have a long-lasting effect.

## Limitations And Potential Explanations

This meta-analysis had several limitations. First, there are many clinical trials in development like NCT03496675 and NCT03271190 (Clinicaltrials.gov), whose completion is estimated to be in 2024 and 2022, respectively, which could not be included in this analysis (56, 57). Secondly, there are several important limitations in the design of the trials included. First, some of the studies included had a very small sample size (<100 participants), which means that they may lack enough participants to detect differences between groups. Also, the musical interventions and the method used to evaluate the cognitive function and depression were diverse and make it difficult to state clearly their benefit when compared to usual care. The lack of standardized methods for musical stimulus selection is a common drawback in the studies we reviewed and a probable contributor to inconsistencies across studies (55).

Finally, we could not perform a subgroup analysis regarding dementia severity to evaluate when music intervention would be more appropriate in the disease trajectory. This was due to the fact that in all studies selected, participants with different dementia stage were randomly assigned to the intervention or control group. Besides, almost all trials in the literature were focused on the mild or moderate stage of dementia, and there were few studies about people living with severe dementia. However, those studies do not evaluate cognitive function (58).

## Future Research Recommendation

Despite the limitations, music is a non-pharmacological intervention, noninvasive, and without side effects, and its application is economical (53, 54). For this reason, in order to confirm the effect of musical interventions, more clinical trials on the effect of music therapy should be promoted. The tests should include a high number of participants, be robust, and be randomized. As explained, music therapy methods and techniques used in clinical practice are diverse. Therefore, it is necessary to design standardized clinical trials that evaluate cognitive function and the disease behavioral features through the same battery of tests to obtain comparable results. On the other hand, there were no high-quality longitudinal studies that demonstrated long-term benefits of music therapy. It is also important to develop study designs that will be sensitive to the nature and severity of dementia. Future music therapy studies need to define a theoretical model, include better-focused outcome measures, and discuss how the findings may improve the well-being of people with dementia as discussed by McDermott et al. (45). and many others (49, 54, 55).

The investment in research in this novel therapy could lead to its implementation as a new and alternative intervention together with current cognitive-behavioral and pharmacological therapies.

## Data Availability Statement

All datasets generated for this study are included in the article/Supplementary Material.

## Author Contributions

CM-M and PM-M: did systematic review and review the manuscript. CM-M and RC: meta-analysis. RC: meta-regression and sub-group analysis and review the manuscript. CP: design the study, conceptualization, supervision, wrote the paper.

## Conflict of Interest

The authors declare that the research was conducted in the absence of any commercial or financial relationships that could be construed as a potential conflict of interest.
