# Supplementary material for: Music Therapy in the Treatment of Dementia: A Systematic Review and Meta-Analysis
Source: Front Med (Lausanne). 2020 May 19;7:160. doi: 10.3389/fmed.2020.00160 (PMC7248378; doi:10.3389/fmed.2020.00160)
Supplement: Supplementary file 1 [file Data_Sheet_1.docx]

**Supplementary data**

- Search procedures
- Supplementary methods

Table 1 and Table 2

- Supplementary tables and figures

Table 1, Table 2, Figure 1

PRISMA Checklist

**Supplementary data**

**SEARCH PROCEDURES**

We conducted an electronic search on Medline, Pubmed Central, EMbase, Psycinfo and Cochrane Library databases, as well as google scholar platform using the terms “music” AND “Brain” finding 4491 articles in PubMed, 4574 in Pubmed Central, 189 articles in the Cochrane library, 237 articles in Psycinfo and 58 articles in EMbase, in total 9549 articles were found. When delimiting the search with the term “Dementia” we found 153 articles in PubMed, 353 in Pubmed Central, 16 articles in Cochrane library, 28 articles in Psycinfo and 7 articles in EMbase, a total of 561 articles. Finally, the search was completed by adding the term “Clinical trial”, finding 11 articles in PubMed, 16 articles in Pubmed Central, 19 articles in Cochrane library, 4 articles in Psychinfo and 0 articles in EMbase, so the search ended with 50 articles.

Two review authors independently assessed publications for eligibility by checking the title and, if available, the abstract. If any doubt referred to an article’s relevance existed, they retrieved and assessed the full article. All studies that compared any form and method of musical intervention with an intervention without music were evaluated. Thus, reviews (n=16), study protocols (n=7) and duplicate articles (n=6) were eliminated, so the articles evaluated full text were 21. Finally, those whose do not evaluate the cognitive function (n=13) were removed. Any disagreements were resolved by discussion and consensus between researchers until 100% agreement was reached. Therefore, we finally stayed for the systematic review and meta-analysis with 8 articles.

**Supplementary methods**

| **PEDro scale** |
| --- |
| 1. Eligibility criteria were specified 2. Subjects were randomly allocated to groups (in a crossover study, subjects were randomly allocated an order in which treatments were received) 3. Allocation was concealed 4. The groups were similar at baseline regarding the most important prognostic indicator 5. There was blinding of all subjects 6. There was blinding of all therapists who administered the therapy 7. There was blinding of all assessors who measured at least one key outcome 8. Measures of at least one of the key outcomes were obtained from more than 85% of the subjects initially allocated to groups 9. All subjects for whom outcome measures were available received the treatment or control condition as allocated or, where this was not the case, data for at least one key outcome was analysed by “intention to treat” 10. The results of between-group statistical comparisons are reported for at least one key outcome 11. The study provides both point measures and measures of variability measures for at least one key outcome |
|  |

**Table 1. PEDro Scale**. It shows the 11 criteria to evaluate the quality of the trials included in the study. For a trial to be considered quality it must obtain a score greater than 5. If the first criterion is not met, the trial is discarded directly. The questions are answered with “Yes” or “No” (de Morton 2009)

**Table 2. CASP scale**. It shows the 11 questions to evaluate a clinical trial, consisting of three major epigraphs. The first three questions are eliminatory: if your answer is “yes”, it is worth continuing with the remaining questions. The questions are answered with

“Yes” or “No” (Hyde *et al.* 2015)

| **CASP scale** |
| --- |
| **Are the test results valid?** |
| 1. Is the trial oriented to a clearly defined question? |
| 1. Was the assignment of patients to treatments random? |
| 1. Were all the patients who entered it properly considered until the end of the study? 2. Was blinding maintained: patients, clinics, study staff? 3. Were the groups similar at the beginning of the trial? 4. Apart from the study intervention, groups were treated in the same way? |
| **What are the results?** |
| 1. Is the effect of the treatment very large? 2. What is the precision of this effect? |
|  |
| **Can these results help us?** |
| 1. Can these results be applied in your local environment or population? 2. Were all results of clinical importance taken into account? 3. Do the benefits to obtain justify the risks and costs? |

| **PEDro scale score** | | | | | | | | | | | | |
| --- | --- | --- | --- | --- | --- | --- | --- | --- | --- | --- | --- | --- |
|  | ***Eligibility criteria specified*** | ***Random allocation*** | ***Concealed allocation*** | ***Comparable at baseline*** | ***Blinded subjects*** | ***Blinded therapists*** | ***Blinded assessors*** | ***Adequate follow-up*** | ***Intention to treat*** | ***Between group comparisons*** | ***Point estimates and variability*** | ***Summary*** |
| **Särkämö et al, 2014** | 1 | 1 | 0 | 0 | 1 | 0 | 0 | 1 | 1 | 1 | 1 | **7** |
| **Särkämö et al, 2016** | 1 | 1 | 0 | 0 | 0 | 0 | 0 | 1 | 1 | 1 | 1 | **6** |
| **Doi et al, 2017** | 1 | 1 | 1 | 0 | 1 | 0 | 0 | 1 | 1 | 1 | 0 | **7** |
| **Han et al, 2017** | 1 | 1 | 0 | 0 | 1 | 1 | 0 | 1 | 1 | 1 | 1 | **8** |
| **Ceccato et al 2012** | 1 | 1 | 1 | 1 | 1 | 1 | 0 | 0 | 1 | 1 | 1 | **9** |
| **Chu et al 2014** | 1 | 1 | 1 | 1 | 1 | 0 | 0 | 1 | 1 | 1 | 1 | **9** |
| **Lyu et al 2018** | 1 | 1 | 0 | 1 | 0 | 0 | 0 | 1 | 1 | 0 | 1 | **6** |
| **Guétin et al 2009** | 1 | 1 | 1 | 1 | 1 | 0 | 0 | 1 | 1 | 0 | 0 | **7** |

**Supplementary tables and figures**

**Supplementary Table 1. PEDro scale score.**  Each criterion is scored after reading the different articles. From a score of 5 on the PEDro scale it is considered that the study has enough quality. The maximum score was 8 and the minimum score was 5. Since it was not known if the studies were comparable at the beginning, the scores for all the studies was 0. Yes=1; No =0

| **CASP scale score** | | | | | | | | | | | | |
| --- | --- | --- | --- | --- | --- | --- | --- | --- | --- | --- | --- | --- |
|  | **Definite question** | **Random allocation** | **Consideration of patients** | **Blinding** | **Comparable to the start** | **Groups treated equally** | **Treatment effect** | **Precise of effect** | **Aplicable to another population** | **Consideration of results of clinical importance** | **Justification benefits/costs and risks** | **Summary** |
| **Särkämö et al, 2014** | 2 | 2 | 2 | 2 | 0 | 2 | 0 | - | 2 | 2 | 2 | **16** |
| **Särkämö et al, 2016** | 2 | 2 | 2 | 0 | 0 | 2 | 0 | - | 2 | 2 | 2 | **14** |
| **Doi et al, 2017** | 2 | 2 | 2 | 2 | 0 | 2 | 0 | - | 2 | 2 | 2 | **16** |
| **Han et al, 2017** | 2 | 2 | 2 | 2 | 0 | 2 | 0 | - | 2 | 2 | 2 | **16** |
| **Ceccato et al. 2012** | 2 | 2 | 2 | 2 | 2 | 2 | 2 | - | 2 | 2 | 2 | **20** |
| **Chu et al 2014** | 2 | 2 | 2 | 2 | 2 | 2 | 0 | - | 2 | 2 | 2 | **18** |
| **Lyu et al 2018** | 2 | 2 | 2 | 2 | 2 | 2 | 0 | - | 2 | 2 | 0 | **16** |
| **Guétin et al 2009** | 2 | 2 | 2 | 2 | 2 | 2 | 0 | - | 2 | 2 | 2 | **18** |

**Supplementary Table 2. CASP scale score.** Each criterion is scored after reading the different articles. From a score of 9 on the CASP scale, the study is considered to be of sufficient quality. The maximum score was 16 and the minimum score was 13. As it was not known if the studies were comparable at the beginning, the scores for all the studies was 0, as well as with the effect of the treatment and, therefore, the accuracy of the effect is also not known. Yes= 2, No=1, Not known= 0


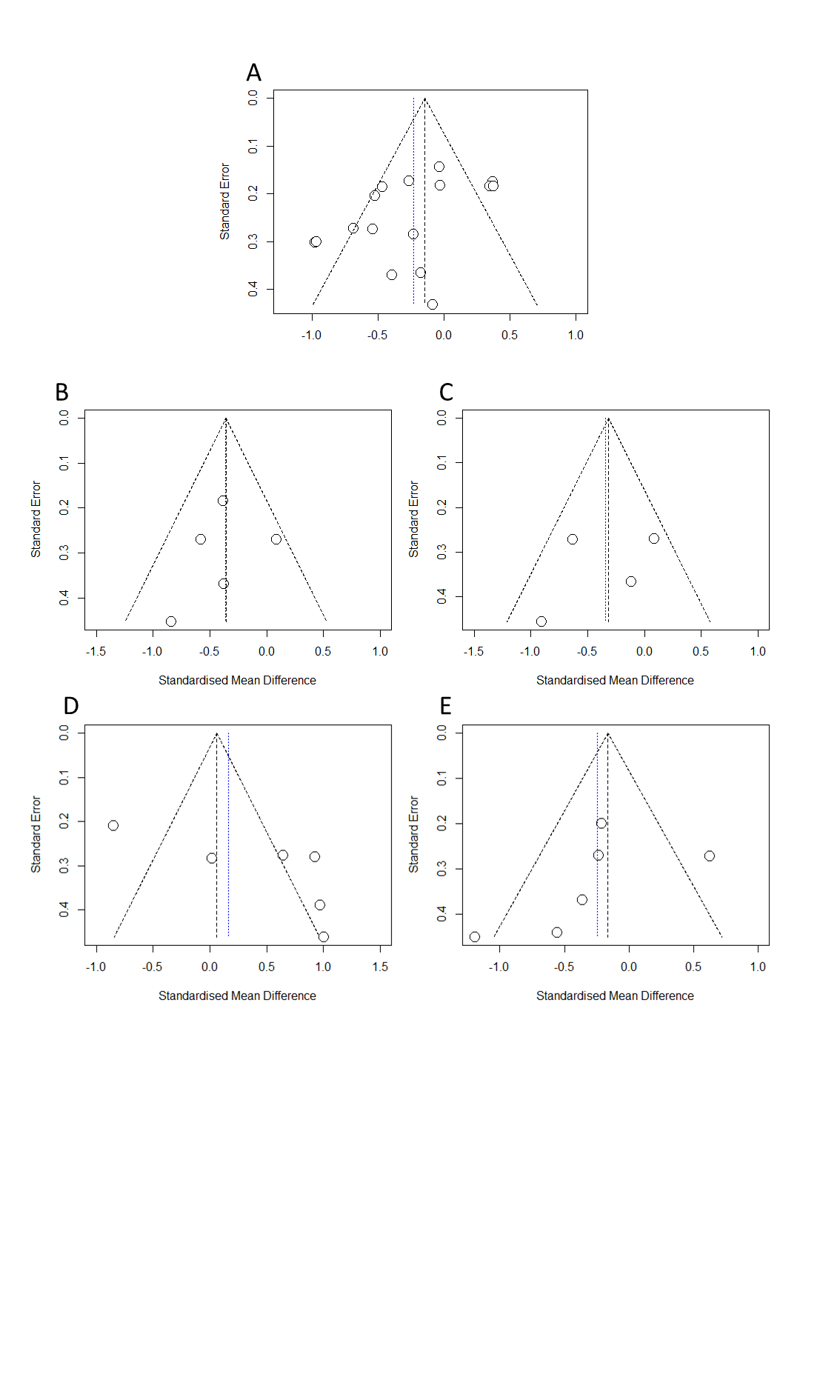


**Supplementary Figure 1: Funnel plots. It represents the publication bias for each of the study included in meta-analysis. A.** Cognitive function **B.** Quality of life **C.** Quality of life at 6 months **D.** Depressive state. **E** Depressive state at 6 months

| **Section/topic** | **#** | **Checklist item** | **Reported on page #** | |  |
| --- | --- | --- | --- | --- | --- |
| **TITLE** | | |  | |  |
| Title | 1 | Identify the report as a systematic review, meta-analysis, or both. | 1 | |  |
| **ABSTRACT** | | |  | |  |
| Structured summary | 2 | Provide a structured summary including, as applicable: background; objectives; data sources; study eligibility criteria, participants, and interventions; study appraisal and synthesis methods; results; limitations; conclusions and implications of key findings; systematic review registration number. | 1 | |  |
| **INTRODUCTION** | | |  | |  |
| Rationale | 3 | Describe the rationale for the review in the context of what is already known. | 2-3 | |  |
| Objectives | 4 | Provide an explicit statement of questions being addressed with reference to participants, interventions, comparisons, outcomes, and study design (PICOS). | 3 | |  |
| **METHODS** | | |  | |  |
| Protocol and registration | 5 | Indicate if a review protocol exists, if and where it can be accessed (e.g., Web address), and, if available, provide registration information including registration number. | No | |  |
| Eligibility criteria | 6 | Specify study characteristics (e.g., PICOS, length of follow-up) and report characteristics (e.g., years considered, language, publication status) used as criteria for eligibility, giving rationale. | 3-4 | |  |
| Information sources | 7 | Describe all information sources (e.g., databases with dates of coverage, contact with study authors to identify additional studies) in the search and date last searched. | 3 | |  |
| Search | 8 | Present full electronic search strategy for at least one database, including any limits used, such that it could be repeated. | Sup. data | |  |
| Study selection | 9 | State the process for selecting studies (i.e., screening, eligibility, included in systematic review, and, if applicable, included in the meta-analysis). | 4-5 | |  |
| Data collection process | 10 | Describe method of data extraction from reports (e.g., piloted forms, independently, in duplicate) and any processes for obtaining and confirming data from investigators. | 4-7 | |  |
| Data items | 11 | List and define all variables for which data were sought (e.g., PICOS, funding sources) and any assumptions and simplifications made. | Table1 | |  |
| Risk of bias in individual studies | 12 | Describe methods used for assessing risk of bias of individual studies (including specification of whether this was done at the study or outcome level), and how this information is to be used in any data synthesis. | 5-6 Sup. data | |  |
| Summary measures | 13 | State the principal summary measures (e.g., risk ratio, difference in means). | 5-6 | |  |
| Synthesis of results | 14 | Describe the methods of handling data and combining results of studies, if done, including measures of consistency (e.g., I^2^) for each meta-analysis. | 6-7 | |  |
| Section/topic | # | Checklist item | Reported on page # | |  |
| Risk of bias across studies | 15 | Specify any assessment of risk of bias that may affect the cumulative evidence (e.g., publication bias, selective reporting within studies). | 6-7 | |  |
| Additional analyses | 16 | Describe methods of additional analyses (e.g., sensitivity or subgroup analyses, meta-regression), if done, indicating which were pre-specified. | 7 | |  |
| **RESULTS** | | |  | |  |
| Study selection | 17 | Give numbers of studies screened, assessed for eligibility, and included in the review, with reasons for exclusions at each stage, ideally with a flow diagram. | | Figure 1 | |
| Study characteristics | 18 | For each study, present characteristics for which data were extracted (e.g., study size, PICOS, follow-up period) and provide the citations. | | Table 1 | |
| Risk of bias within studies | 19 | Present data on risk of bias of each study and, if available, any outcome level assessment (see item 12). | | Figure 1 Sup.data | |
| Results of individual studies | 20 | For all outcomes considered (benefits or harms), present, for each study: (a) simple summary data for each intervention group (b) effect estimates and confidence intervals, ideally with a forest plot. | | Figure 2 | |
| Synthesis of results | 21 | Present results of each meta-analysis done, including confidence intervals and measures of consistency. | | 7-9 | |
| Risk of bias across studies | 22 | Present results of any assessment of risk of bias across studies (see Item 15). | | Table 2 | |
| Additional analysis | 23 | Give results of additional analyses, if done (e.g., sensitivity or subgroup analyses, meta-regression [see Item 16]). | | 7, Table 2 and Figure 2, | |
| **DISCUSSION** | | |  | |  |
| Summary of evidence | 24 | Summarize the main findings including the strength of evidence for each main outcome; consider their relevance to key groups (e.g., healthcare providers, users, and policy makers). | | 9-12 | |
| Limitations | 25 | Discuss limitations at study and outcome level (e.g., risk of bias), and at review-level (e.g., incomplete retrieval of identified research, reporting bias). | | 12 | |
| Conclusions | 26 | Provide a general interpretation of the results in the context of other evidence, and implications for future research. | | 12-13 | |
| **FUNDING** | | |  | |  |
| Funding | 27 | Describe sources of funding for the systematic review and other support (e.g., supply of data); role of funders for the systematic review. | | N/A | |

*From:*  Moher D, Liberati A, Tetzlaff J, Altman DG, The PRISMA Group (2009). Preferred Reporting Items for Systematic Reviews and Meta-Analyses: The PRISMA Statement. PLoS Med 6(7): e1000097. doi:10.1371/journal.pmed1000097 .
